# Chlorogenic Acid Protects against Atherosclerosis in ApoE^−/−^ Mice and Promotes Cholesterol Efflux from RAW264.7 Macrophages

**DOI:** 10.1371/journal.pone.0095452

**Published:** 2014-09-04

**Authors:** Chongming Wu, Hong Luan, Xue Zhang, Shuai Wang, Xiaopo Zhang, Xiaobo Sun, Peng Guo

**Affiliations:** Pharmacology and Toxicology Research Center, Institute of Medicinal Plant Development, Chinese Academy of Medical Sciences & Peking Union Medical College, Beijing, China; University of Padova, Italy

## Abstract

Chlorogenic acid (CGA) is one of the most abundant polyphenols in the human diet and is suggested to be a potential antiatherosclerotic agent due to its proposed hypolipidemic, anti-inflammatory and antioxidative properties. The aim of this study was to evaluate the effect of CGA on atherosclerosis development in ApoE^−/−^ mice and its potential mechanism. ApoE^−/−^ mice were fed a cholesterol-rich diet without (control) or with CGA (200 and 400 mg/kg) or atorvastatin (4 mg/kg) for 12 weeks. During the study plasma lipid and inflammatory parameters were determined. Treatment with CGA (400 mg/kg) reduced atherosclerotic lesion area and vascular dilatation in the aortic root, comparable to atorvastatin. CGA (400 mg/kg) also significantly decreased plasma levels of total cholesterol, triglycerides and low-density lipoprotein-cholesterol as well as inflammatory markers. Supplementation with CGA or CGA metabolites-containing serum suppressed oxidized low-density lipoprotein (oxLDL)-induced lipid accumulation and stimulated cholesterol efflux from RAW264.7 cells. CGA significantly increased the mRNA levels of PPARγ, LXRα, ABCA1 and ABCG1 as well as the transcriptional activity of PPARγ. Cholesterol efflux assay showed that three major metabolites, caffeic, ferulic and gallic acids, significantly stimulated cholesterol efflux from RAW264.7 cells. These results suggest that CGA potently reduces atherosclerosis development in ApoE^−/−^ mice and promotes cholesterol efflux from RAW264.7 macrophages. Caffeic, ferulic and gallic acids may be the potential active compounds accounting for the *in vivo* effect of CGA.

## Introduction

Atherosclerosis is a major cause of mortality and morbidity and is the single most important cause of cardiovascular disease (CVD) [1], [2]. Dyslipidemia is a well-recognized risk factor for atherosclerosis [3], [4]. Currently, a popular approach for the treatment of atherosclerosis is to reduce plasma lipid levels for example by using statins. However, statin use prevents only 50%–60% of all cardiovascular events [5]. As atherosclerosis is considered as a multifactorial inflammatory disease and inflammation, oxidative stress, and macrophage foam cell formation are crucial processes in the development of atherosclerotic plaques [6], optimal therapeutic treatment of atherosclerosis should therefore encompass different approaches.

Macrophage foam cell formation is a key determinant of atherosclerotic lesion occurrence [7]. Multiple investigations have demonstrated that inhibition of macrophage foam cell formation by stimulating cholesterol efflux can efficiently prevent atherosclerotic plaque occurrence [8], [9], [10]. In the regulation of cholesterol efflux, ATP-binding cassette transporters A1/G1 (ABCA1/ABCG1) play pivotal roles [3]. ABCA1 promotes the efflux of cholesterol to lipid-poor apolipoproteins such as apoA1 while ABCG1 has a critical role in mediating cholesterol efflux to high-density lipoprotein (HDL) [11]. Recent studies have shown that agonists of peroxisome proliferator-activated receptor γ (PPARγ) can stimulate cholesterol efflux via upregulating the expression of ABCA1, which is mediated by liver X receptor α (LXRα) [10], [12]. Currently, the PPARγ-LXRα-ABCA1 pathway has been deemed as an important target for the prevention and treatment of atherosclerosis [10], [13].

Chlorogenic acid (CGA, 5-caffeoylquinic acid) is one of the most abundant polyphenols in the human diet, which can be found in carrot, tomato, sweet potato, apple, peach, prune, oilseeds and coffee [14]. Like other dietary polyphenols, CGA has numerous nutritional and pharmacological activities such as antidiabetes [15], antihypertension [16] and antitumor [17]. Importantly, CGA has also been recognized to possess various antiatherosclerotic activities, including hypolipidemic [18], [19], antioxidative [20], [21] and anti-inflammatory [22], [23] properties. Despite these promising and diverse antiatherosclerotic actions, investigations addressing the effect of CGA on atherosclerosis are scarce. Recent preliminary reports suggest that CGA indeed reduces atherosclerosis development [24]. In the current study, we evaluated whether CGA protects against atherosclerosis development in ApoE^−/−^ mice fed a cholesterol-rich diet. The effect and potential mechanisms of CGA on chlesterol efflux from macrophages were also investigated.

## Materials and Methods

### Ethics statement

All animal experiments were approved by the Medical Ethics Committee of Peking Union Medical College and were in accordance with the National Institutes of Health regulations for the care and use of animals in research. All efforts were made to minimize suffering.

### Reagents

Chlorogenic acid which was isolated from the flower of *Lonicera japonica* Thunb. and with a purity ≥98% was purchased from National Institutes for Food and Drug Control (Beijing, China). Caffeic, quininic, ferulic, gallic and vanillic acids were purchased from Sigma-Aldrich (Shanghai, China). Atorvastatin and lipopolysaccharides (LPS) were purchased from Sigma-Aldrich Co. Ltd. (St. Louis, USA). 2′,7′-Dichlorodihydrofluorescein diacetate (DCFH-DA) was obtained from Invitrogen (Shanghai, China).

### Animals and Treatment

Male C57BL/6J ApoE^−/−^ mice (6–8 weeks old), weighing 20–25 g, were purchased from Vital River Laboratory Animal Technology Co., Ltd.(Beijing, China). The animals were kept in a humidity-controlled room on a 12-h light–dark cycle with food and water available *ad libitum* for one week. The mice were then divided randomly into four groups with six animals in each group and fed a high-fat diet (78.8% standard diet, 10.0% yolk powder, 10.0% lard, 1.0% cholesterol, and 0.2% sodium taurocholate) for 12 weeks. The control group (ApoE^−/−^ group) was given equal volumn of distilled water while the ApoE^−/−^ + atorvastatin, ApoE^−/−^ + CGA-200, and ApoE^−/−^ + CGA-400 groups were administrated by oral gavage with atorvastatin (4 mg/kg) [25] or CGA (200 or 400 mg/kg) respectively. At the end of the 12-week period, after the animals were fasted overnight, blood samples were collected for estimation of plasma levels of lipids and inflammatory factors by kits (Jian Cheng Biotechnology Company, Nanjing, China). Animals were then euthanized, and the aorta roots were collected, fixed with 4% formaldehyde for 24 h, and embedded in paraffin. The aorta root was serially sectioned in 6-μm sections and 6 consecutive sections were stained with hemalaune and erythrosine (H&E) for atherosclerotic plaque evaluation. Images were captured with a Zeiss Axio Camera (Carl Zeiss, Jena, Germany). The plaque area and plaque coverage percentage of the total vessel surface area were measured using ImageJ software.

### *In vivo* ultrasound

After treated with CGA for 11 weeks, mice were anesthetized with inhaled 1–2% isoflurane titrated to a heart rate of 470–500 beats per minute and shaved. The ascending aorta was visualized in one plane from the aortic valve to the transverse aorta in a parasternal long axis view using a 40 MHz high frequency Visual Sonics Vevo 660 ultrasound machine. The diameter of the ascending aorta 2 cm above the sinus of Valsalva and the diameter of the proximal innominate artery (brachiocephalic artery) was measured by the leading edge method.

### Cell culture

RAW264.7 cells, which originated from the American Type Culture Collection (ATCC) (Manassas, VA, USA), were obtained from the Peking Union Medical College. Cells were maintained in DMEM medium (Gibco, Grand Island, NY, USA) supplemented with 10% fetal bovine serum (Gibco), penicillin (100 U/mL) and streptomycin (100 µg/mL) at 37°C in 5% CO_2_. When grown to 70%–80% confluence, cells were incubated in DMEM supplemented with oxLDL (50 mg/mL, Xiesheng Biotechnologies, Beijing, China) and indicated concentration of CGA for 24 hours. Subsequently, the cells were subjected to oil-red O staining or total cholesterol determination as described previously [26].

### Oil red O staining

Lipid staining was assessed histologically using oil red O staining. Treated RAW 264.7 cells were incubated with oxLDL (50 mg/mL) in medium containing lipoprotein-deficient human serum for 24 h. Cells were then fixed with 4% w/v paraformaldehyde (30 min, room temperature) and stained with filtered oil red O solution (60 min, room temperature). The staining was evaluated by both microscopic examination (Olympus, Tokyo, Japan) and spectrophotometry at 358 nm.

### Measurement of cholesterol in macrophages

The concentration of intracellular cholesterol was determined by kits as previously reported [27]. The protein pellet was solubilized in 1 mol/L NaOH and protein concentration was determined by the BCA Protein Assay (Thermo Fisher Scientific Inc. IL, USA).

### Measurement of IL-1β, IL-6 and TNF-α in RAW264.7 cells

For the measurement of IL-1β, IL-6 and TNF-α, RAW264.7 cells were treated with CGA in the presence or absence of LPS (1 µg/ml) for 24 h. IL-1β, IL-6 and TNF-α were assayed using the ELISA kits according to the manufacturer's instructions (Jian Cheng Biotechnology Company, Nanjing, China).

### Cholesterol efflux assay

RAW264.7 cells were equilibrated with NBD-cholesterol (1 µg/mL) for 12 h. NBD-cholesterol-labeled cells were washed with PBS and incubated in serm-free DMEM medium containing 50 µg/mL HDL or ApoA1 and indicated concentration of respective compound for 6 h. The fluorescence-labeled cholesterol released from cells into the medium was measured with a Tecan Infinite M1000Pro Microplate Reader (TECAN Group Ltd, Shanghai, China). Cholesterol efflux was expressed as a percentage of fluorescence in the medium relative to the total amounts of fluorescence detected in cells and the medium. Each experiment was performed in triplicate with 3 replicates each.

### Serum pharmacology on RAW264.7 cells

Normal male C57BL/6J mice were orally gavaged with 400 mg/kg of CGA or equal volume of distilled water for 3 days. Blood was collected at 45 min after the final treatment and serum was prepared by centrifugation at 3500 rpm for 15 min. oxLDL-induced lipid accumulation and cholesterol efflux assay on RAW264.7 cells were performed as described above. Equal volume of serum (20 µL in 2 mL medium) from animals treated with CGA (S_CGA_) or distilled water (S_NC_) was used for the experiment.

### Measurement of PPARγ promoter activities

Transactivation reporter assay in 293T cells was performed as previously described [28]. Briefly, cells were transiently transfected with PPARγ expression vector and DR-1 luciferase reporter vector. At 6 h after transfection, the transfection mixture was replaced with fresh medium containing the appropriate agonist. Luciferase assays were performed after 24 h using luciferase assay kit (Promega, Beijing, China) according manufacturer's instruction.

### Realtime quantitative PCR

Total RNA extraction, cDNA synthesis and quantitative PCR assays were performed as described previously [29]. The normalized expression levels of the target genes were estimated as described previously [30]. At least three independent biological replicates were performed to check the reproducibility of the data. The gene-specific primers used for quantitative PCR are listed in Table S1.

### Statistical analyses

Data are presented as mean±SEM. Differences were assessed by one-way analysis of variance (ANOVA) test followed by the Dunnett's post hoc test. Two-way analysis of covariance (ANCOVA) was performed to test for differences on atherosclerotic lesion area after controlling for the cholesterol-lowering capacity of the different treatments. The square root was taken of the atherosclerotic lesion area to linearize the relationship with plasma cholesterol exposure. A probability level (P) of 0.05 was considered significant. The Student's t test was used to evaluate differences in the in vitro macrophage studies. SPSS 17.0 for Windows (SPSS, Chicago, IL, USA) was used for statistical analysis.

## Results

### CGA attenuates atherosclerosis development

**Figure 1:**
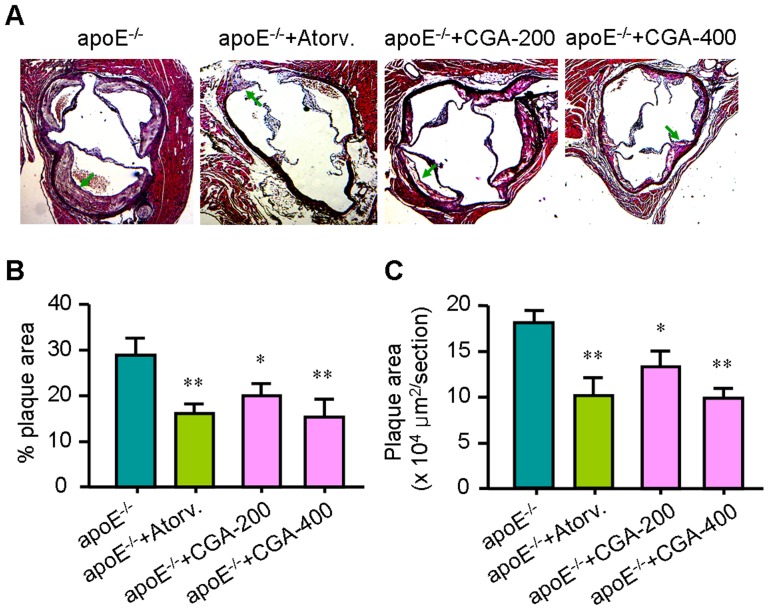
Chlorogenic acid (CGA) reduces atherosclerosis development. Slides of the valve area of the aortic root were stained with hematoxylin and eosin (H&E) (A), and plaque coverage percentage of the total vessel surface area (B) and lesion area (C) were calculated in four sections per mouse starting from the appearance of open aortic valve leaflets. Values are means±SEM (n = 24). *p<0.05, **p<0.01 vs. ApoE^−/−^ group. Atorv.  =  atorvastatin, CGA  =  Chlorogenic acid.

To study the effect of CGA on atherosclerosis development, ApoE^−/−^ mice were fed a cholesterol-rich diet without or with CGA or atorvastatin. Neither of the treatments affected food intake and body weight during the study (not shown). Mice were sacrificed after 12 weeks of treatment, and lesion size was determined in the valve area of the aortic root. The atherosclerotic lesions were indicated by arrows in Figure 1A. As shown in Figure 1, treatment with CGA (400 mg/kg) reduced the percentage and the total atherosclerotic lesion area by 44.1% and 51.7%, respectively (*P*<0.01), whereas atorvastatin reduced this by 42.3% and 49.6%, respectively (*P*<0.01) as compared to control treated mice.

### CGA reduces aortic dilatation

**Figure 2:**
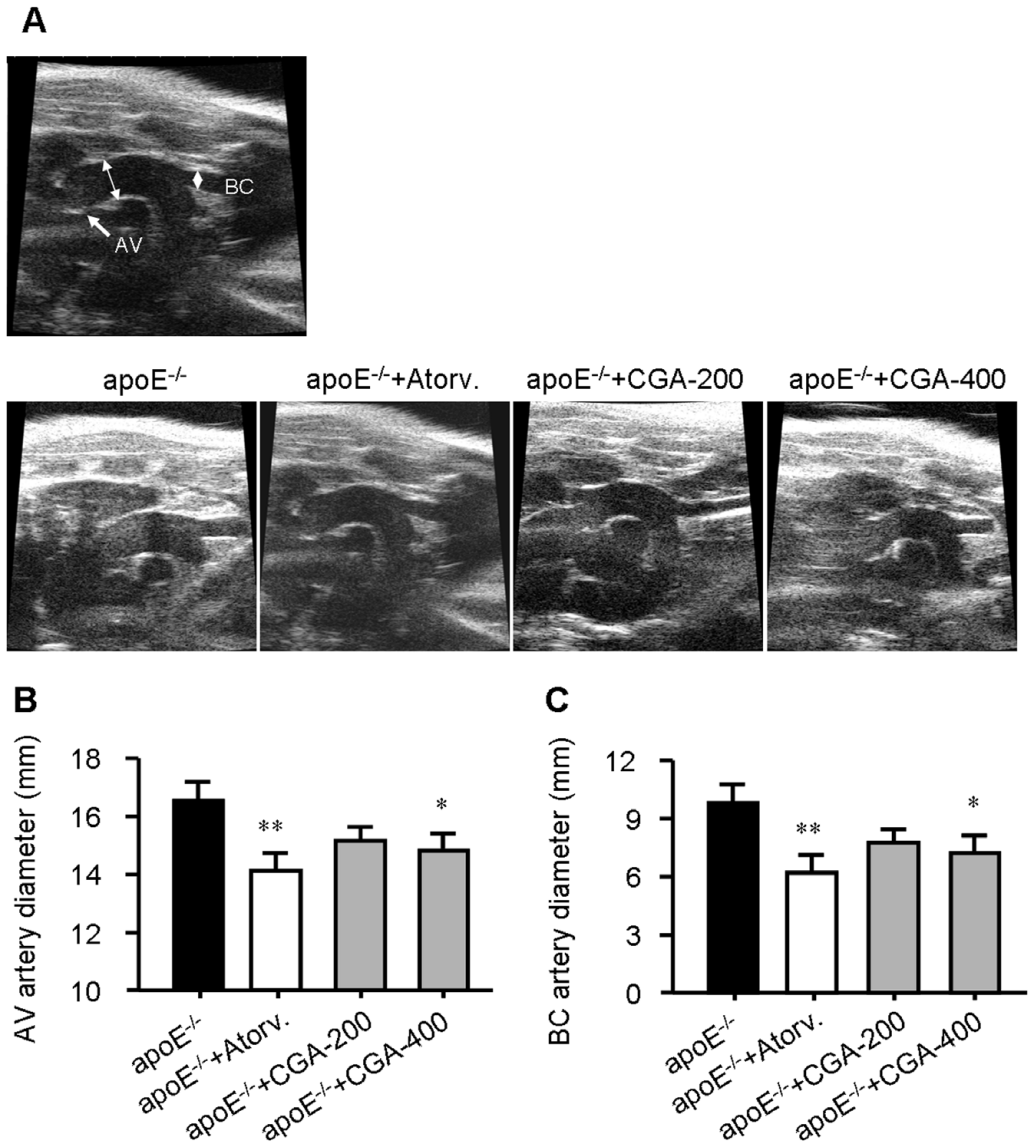
Treatment with Chlorogenic acid (CGA) reduces aortic dilatation. (A) In vivo ultrasound for measurement of the diameter of the ascending aorta 2 cm above the aortic valve (AV, arrow) and at the origin of the brachiocephalic (BC) artery were quantified in B and C (n  = 6). Values are means±SEM. *p<0.05, **p<0.01 vs. ApoE^−/−^ group. Atorv.  =  atorvastatin, CGA  =  Chlorogenic acid.

We also measured the vascular lumen diameter using high frequency ultrasound to assess the effect of CGA on aortic dilatation. Treatment with CGA (400 mg/kg) or atorvastatin (4 mg/kg) for 12 weeks significantly reduced vascular wall thickness of the ascending aorta 2 cm above the aortic valve (AV, arrow) and at the origin of the brachiocephalic (BC) artery (Figure 2), suggesting that CGA inhibits aortic dilatation in ApoE^−/−^ mice.

### CGA decreases the serum levels of total cholesterol (TC), low-densitiy lipoprotein-cholesterol (LDL-c) and triglyceride (TG)

Dyslipidemia is a well-recognized risk factor for atherosclerosis, we therefore investigated the effect of CGA on serum lipids levels. Compared with the control (ApoE^−/−^) group, the serum levels of TC, and LDL-c were siginificantly decreased in the ApoE^−/−^ + atorvastatin (4 mg/kg) group (*P*<0.01) and the ApoE^−/−^ + CGA (400 mg/kg) group (*P*<0.05). Treatment with CGA (400 mg/kg) also significantly reduced the serum TG level (*P*<0.05) while atorvastatin only exerted a non-significant reduction on TG (Table 1). Atorvastatin and CGA increased the serum level of high-density lipoprotein-cholesterol (HDL-c) but their effects were not statistically significant.

**Table 1:** The serum lipid profile.

	TC (mmol/L)	TG (mmol/L)	HDL-c (mmol/L)	LDL-c (mmol/L)
ApoE−/−	14.32±1.34	2.30±0.46	0.82±0.10	3.17±0.39
ApoE−/−+Atorva	11.21±0.89**	1.88±0.27	0.97±0.21	2.45±0.38**
ApoE−/−+CGA-200	12.77±1.67	1.92±0.31	0.76±0.16	2.84±0.44
ApoE−/−+CGA-400	12.11±1.15*	1.69±0.43*	0.88±0.17	2.66±0.41*

TC: total cholesterol; TG: triglyceride; HDL-c: high-density lipoprotein-cholesterol; LDL-c; low-density lipoprotein-cholesterol; Atorva: atorvastatin; CGA: chlorogenic acid.

^*^ *p*<0.05.

^**^ *p*<0.01 v.s. ApoE.

−/− mice (n = 6).

### CGA reduces the levels of proinflammatory cytokines *in vivo* and *in vitro*

**Figure 3:**
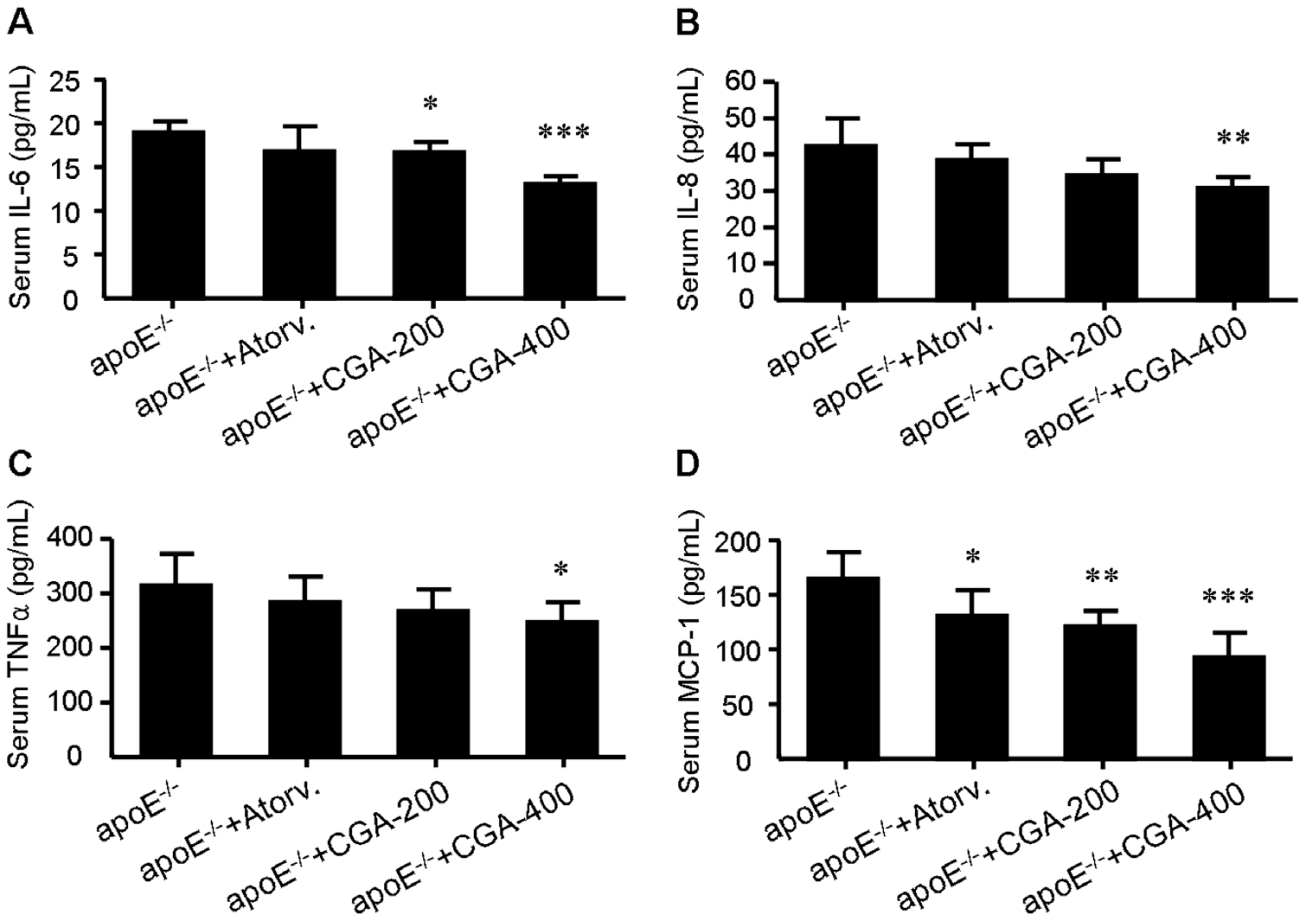
Treatment with chlorogenic acid (CGA) for 12 weeks reduces the serum levels of interleukin-6 (IL-6) (A), interleukin-8 (IL-8) (B), tumor necrosis factor α (TNFα) (C) and monocyte chemotactic protein-1 (MCP-1) (D) in ApoE^−/−^ mice. Values are means±SEM. *p<0.05, **p<0.01, ***p<0.001 vs. ApoE^−/−^ group. Atorv.  =  atorvastatin, CGA  =  Chlorogenic acid.

**Figure 4:**
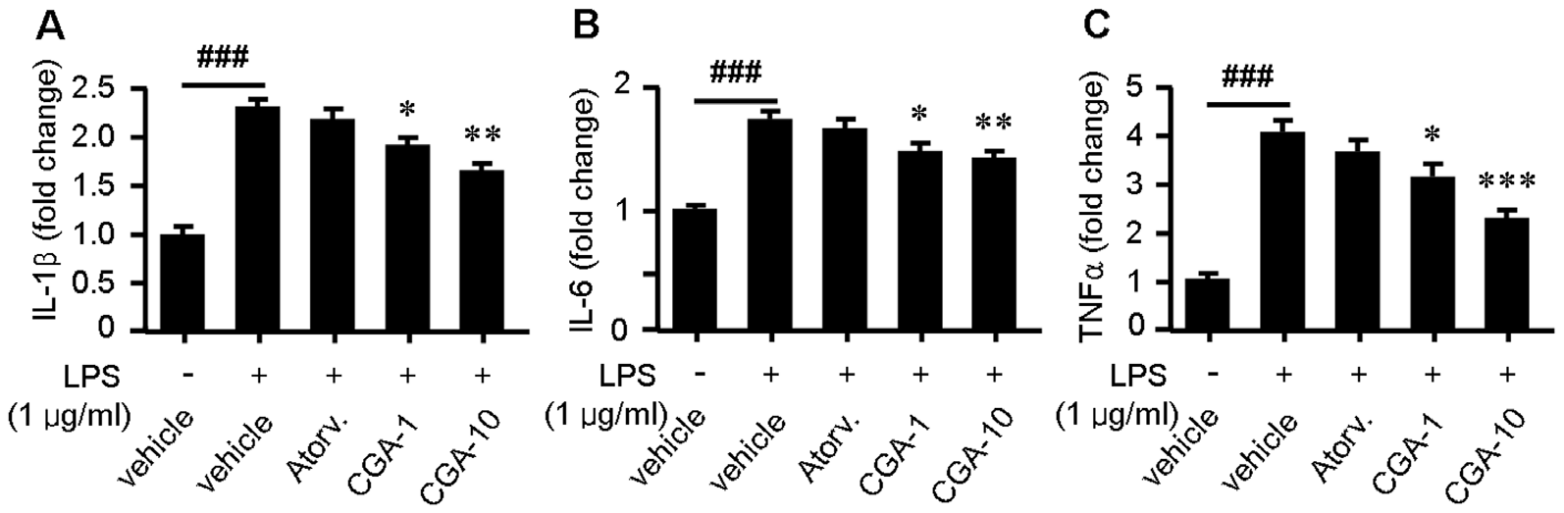
Treatment with chlorogenic acid (CGA) reduces intracellular levels of IL-1β (A), IL-6 (B) and TNFα (C) elicited by LPS in RAW264.7 cells. Values are means ± SEM of at least three experiments. ^###^ *p*<0.001 LPS+vehicle group vs. vehicle group; *p<0.05, **p<0.01, ***p<0.001 test group vs. LPS+vehicle group. Atorv.  =  atorvastatin, CGA  =  Chlorogenic acid, LPS  =  lipopolysaccharides.

As noted previously, CGA pocesses strong anti-inflammatory activity [31]. It is thus of interest to determine whether a similar effect might occur in ApoE^−/−^ mice treated with CGA. As shown in Figure 3, treatment with CGA (400 mg/kg) significantly suppressed serum levels of serum interleukin-6 (IL-6), interleukin-8 (IL-8), tumor necrosis factor α (TNFα) and monocyte chemotactic protein-1 (MCP-1) while administration with atorvastatin (4 mg/kg) showed no significant effect on these inflammatory factors except MCP-1. Similarly, CGA (1 and 10 µM) inhibited LPS-elicited upregulation of IL-1β, IL-8 and TNFα in RAW264.7 macrophages while atorvastatin (1 µM) showed no significant effect (Figure 4).

### CGA inhibits oxidized low-density lipoprotein (oxLDL)-elicited foam cell formation in RAW264.7 cells

**Figure 5:**
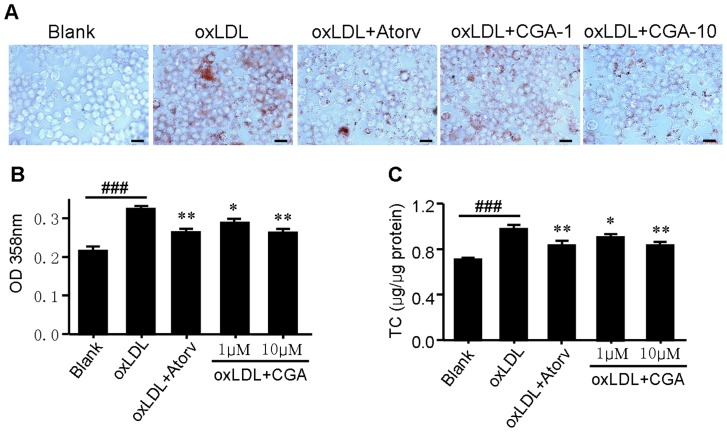
Chlorogenic acid (CGA) inhibits oxidized low-density lipoprotein (oxLDL)-elicited foam cell formation in RAW264.7 cells. RAW264.7 cells were elicited by oxLDL for 24 h with or without supplementation of CGA or atorvastatin. Cells were then stained with Oil red O, and the representative staining pictures (A), the aborptance at 358 nm (B), and intracellular totale cholesterol content (C) were acquired. Bar  =  50 µm. Values are means ± SEM of at least three experiments. ^###^ *p*<0.001 oxLDL group vs. blank group; *p<0.05, **p<0.01 test group vs. oxLDL group. Atorv.  =  atorvastatin, CGA  =  Chlorogenic acid,

The uptake of oxLDL by macrophage induces foam cell formation and promotes the development of atherosclerosis [32]. To determine the effects of CGA on oxLDL induced foam cell formation, we performed oil red O staining and intracellular total cholesterol quantification. RAW264.7 macrophages were incubated with oxLDL (50 mg/mL) for 24 h. The addition of oxLDL to the culture medium induced the foam cell formation as the cytoplasmic lipid accumulation was increased (Figure 5A and 5B). Treatment with CGA and atorvastatin markedly decreased oxLDL-elicited neutral lipid and cholesterol accumulation in RAW264.7 cells (Figure 5A–5C). The results suggested that CGA prevents oxLDL induced foam cell formation in RAW264.7 cells.

### CGA stimulates cholesterol efflux from RAW264.7 cells

**Figure 6:**
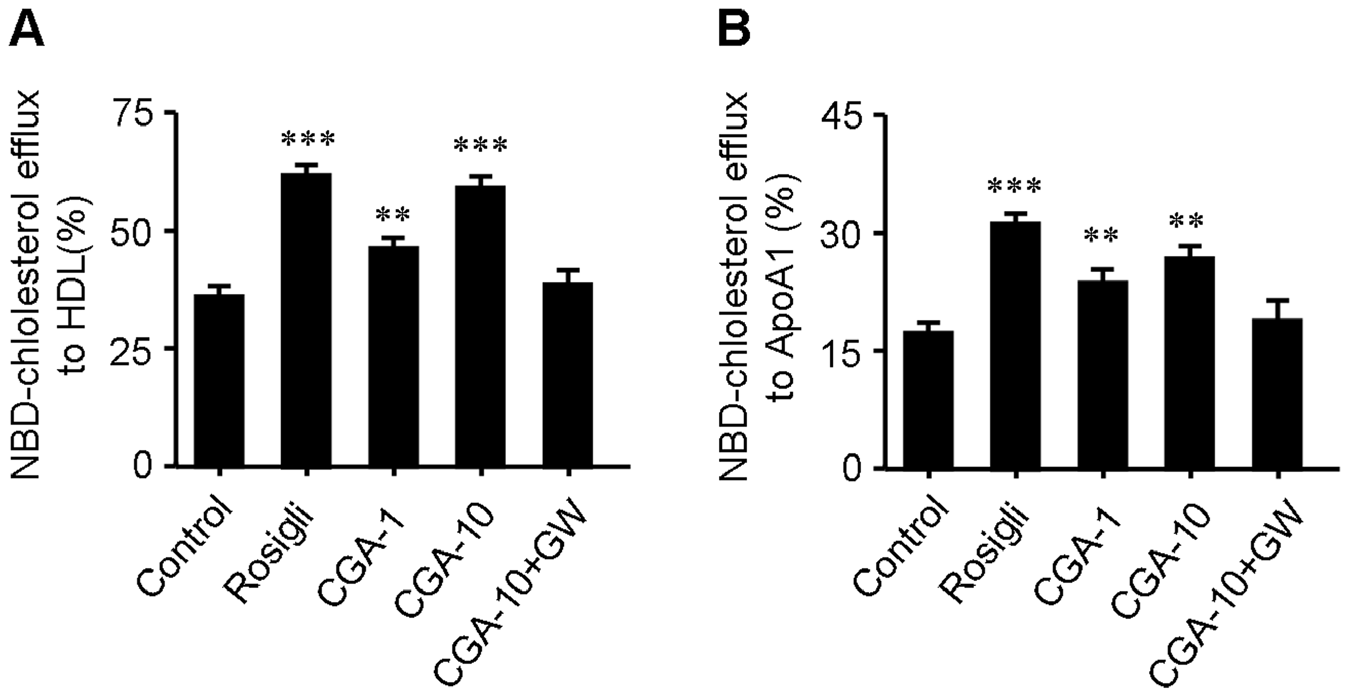
Chlorogenic acid (CGA) stimulates NBD-cholesterol efflux to HDL (A) and ApoA1 (B) in RAW264.7 macrophages. Cells were equilibrated with NBD-cholesterol for 12 h then incubated in serum-free DMEM medium containing HDL or ApoA1 and indicated concentration of CGA for 6 h. Cholesterol efflux was expressed as a percentage of fluorescence in the medium relative to the total amounts of fluorescence detected in cells and the medium. Rosiglitazone (5 µM) was used as positive control while PPARγ inhibitor GW-9662 (20 µM) was used to test the role of PPARγ in CGA-elicited cholesterol efflux. Values are means ± SEM of at least three experiments. *p<0.05, **p<0.01, ***p<0.001 vs. control. Rosigli  =  rosiglitazone, CGA  =  Chlorogenic acid, GW  =  GW-9662.

Promoting cholesterol efflux is an effective approach to suppress foam cell formation. We evaluated the effect of CGA on cholesterol efflux from macrophages by a NBD-cholesterol efflux assay. As shown in Figure 6, treatment with CGA (10 µM) increased NBD-cholesterol efflux to HDL by ∼38% and increased NBD-cholesterol efflux to ApoA1 by ∼33% which is comparable to rosiglitazone (5 µM, ∼35% and ∼47%, respectively), a popular PPARγ agonist and known to promote cholesterol efflux [33]. The promoting effect of CGA on cholesterol efflux in RAW264.7 macrophage was substantially abolished in the presence of GW-9662 (20 µM), an antagonist of PPARγ [34] (Figure 6).

### CGA increases transcription of PPARγ, LXRα, ABCA1 and ABCG1 and upregulates the transcriptional activity of PPARγ

**Figure 7:**
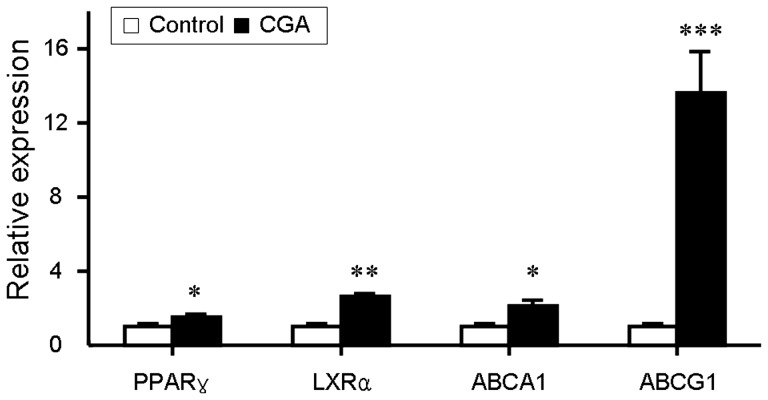
Chlorogenic acid (CGA) upregulates the transcriptional expression of PPARγ, LXRα, ABCA1 and ABCG1 in RAW264.7 cells. Real-time PCR was conducted with gene specific oligonucleotide primers. The amplification of β-actin served as the internal control. Values are means ± SEM of at least three experiments. *p<0.05, **p<0.01, ***p<0.001 vs. control.

**Figure 8:**
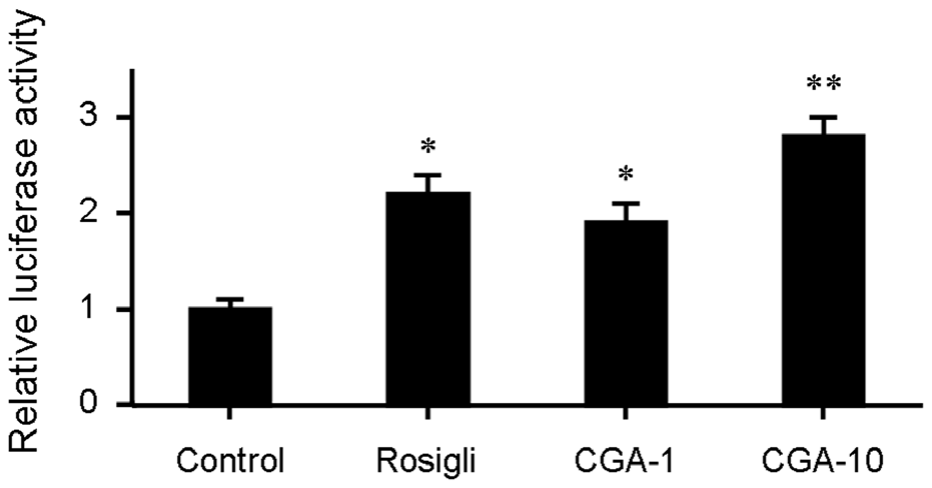
Chlorogenic acid (CGA) increases the transcriptional activity of PPARγ. The transcriptional activity of PPARγ was assessed by transactivation reporter assay in 293T cells. Rosiglitazone (5 µM) was used as positive control. Values are means ± SEM of at least three experiments. *p<0.05, **p<0.01 vs. control. Rosigli  =  rosiglitazone, CGA  =  Chlorogenic acid.

ABCA1and ABCG1 are two pivotal factors in cholesterol efflux which expression is regulated by nuclear trascriptional factors PPARγ and LXRα [12]. We quantified the mRNA levels of PPARγ, LXRα, ABCA1 and ABCG1 by realtime quantitative PCR. Treatment with CGA significantly increased the transcription of PPARγ, LXRα, ABCA1 and ABCG1 (Figure 7). Transactivation reporter assay further showed that CGA increased the transcriptional activity of PPARγ in a dose dependent manner (Figure 8). These results suggested that CGA may stimulate cholesterol efflux through upregulating the expression of PPARγ, LXRα, ABCA1 and ABCG1 and the transcriptional activity of PPARγ.

### CGA metabolites-containing serum inhibits oxLDL-induced lipid accumulation and stimulates cholesterol efflux from RAW264.7 cells

**Figure 9:**
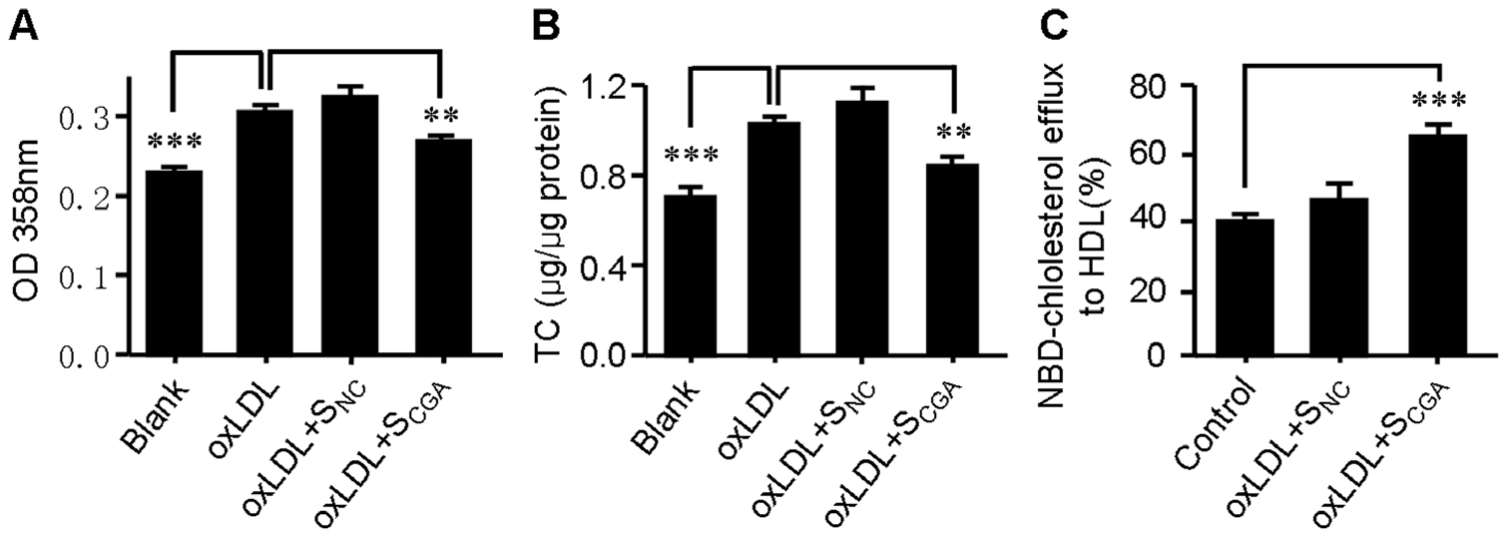
Chlorogenic acid (CGA) metabolites-containing serum from CGA-treated normal mice decreases lipid accumulation and stimulates cholesterol efflux from RAW264.7 cells. Normal C57BL/6J mice were orally gavaged with 400 mg/kg of CGA or equal volume of distilled water for 3 days and blood was collected at 45 min after the final treatment. 1% (v/v) of serum from CGA-treated normal mice (S_CGA_) significantly decreased oxLDL-induced neutral lipid accumulation (A) and total cholesterol (B), and stimulates cholesterol efflux (C) in RAW264.7 cells as compared with that from distilled water-treated animals (S_NC_). Values are means ± SEM of at least three experiments. **p<0.01, ***p<0.001.

Although it has been broadly reported that CGA remained detectable in plasma for at least 6 hours after oral administration [35], [36], it undergoes extensive metabolism during this period. It is possible that the *in vivo* antiatherosclerotic effect of CGA may be mediated by its metabolites rather than CGA itself. To establish the physiological relevance of CGA with the *in vitro* effect on RAW264.7 macrophages, serum pharmacological experiments were conducted. The RAW264.7 cells were treated with equal volume (20 µL in 2 mL medium) of serum obtained from CGA-treated (S_CGA_) or distilled water-treated (S_NC_) mice. As shown in Figure 9, the serum from CGA-treated animals (S_CGA_) significantly inhibited oxLDL-induced lipid accumlation (Figure 9A and 9B) and stimulated cholesterol efflux from RAW264.7 cells (Figure 9C), while serum from distilled water-treated animals (S_NC_) showed no significant influence.

**Figure 10:**
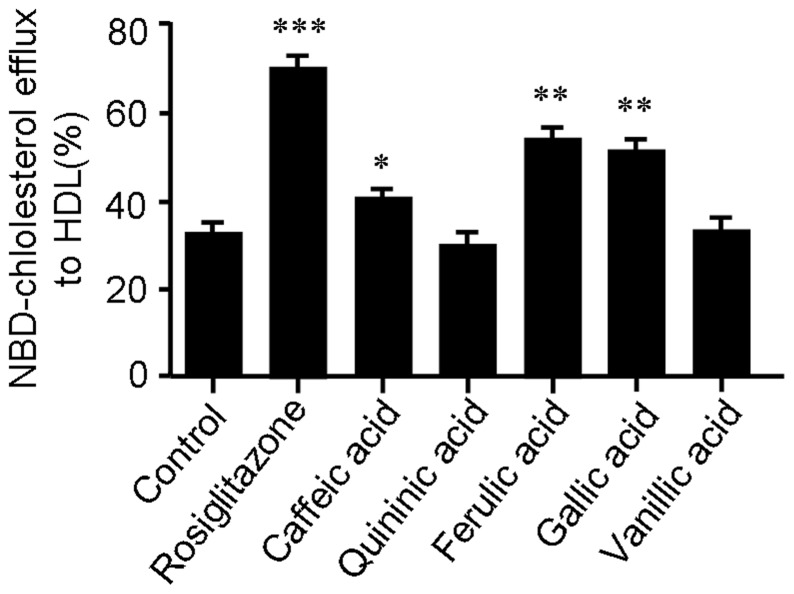
Caffeic acid, ferulic acid and gallic acid are active in promoting cholesterol efflux from RAW264.7 cell mediated by HDL. Cells were equilibrated with NBD-cholesterol for 12 h then incubated in serum-free DMEM medium containing HDL and 10 µM of respective compound for 6 h. Cholesterol efflux was expressed as a percentage of fluorescence in the medium relative to the total amounts of fluorescence detected in cells and the medium. Rosiglitazone (5 µM) was used as positive control. Values are means ± SEM of at least three experiments. *p<0.05, **p<0.01, ***p<0.001 vs. control. Rosigli  =  rosiglitazone.

To further investigate the potential active compounds accounting for the *in vivo* effect of CGA, five key metabolites of CGA, *i.e.* caffeic, quininic, ferulic, gallic and vanillic acids, were tested for the effect on cholesterol efflux. As shown in Figure 10, caffeic, ferulic and gallic acids significantly stimulated cholesterol efflux from RAW264.7 cells mediated by HDL, suggesting that these three metabolites of CGA may be the potential active compounds accounting for the *in vivo* effect of CGA.

## Discussion

Chlorogenic acid is a major phenolic compound in human foods including coffee, fruits and vegetables. Although CGA has been demonstrated to possess various antiatherosclerotic activities, investigations addressing the effect of CGA on atherosclerosis are scarce. Recently, Loke et al [24] reported that daily adminstration of CGA (2 mg/day/mice) helped to diminish atherosclerotic lesion formation in ApoE^−/−^ mice but their differences were not statistically significant. As the daily intake of CGA by coffee drinkers ranges from 500–1000 mg [37], the non-significant effect of CGA on atherosclerotic lesion formation may be due to the low dasage. Therefore, in the present study, we assessed the antiatherosclerotic potential of CGA in ApoE^−/−^ mice at higher doses (200 and 400 mg/kg, corresponding to 5 and 10 mg/day/mice).

Our results showed that CGA (400 mg/kg) significantly reduced atherosclerosis development and prevent aortic dilatation to a similar extent as atorvastatin (Figures 1 and 2) in ApoE^−/−^ mice. As previously reported, treatment with CGA decreased the serum levels of TC, TG and LDL-c, indicating potent hyplipidemic effects (Table 1). To verify whether the antiatherogenic effect of CGA and atorvastatin mainly depended on their ability to reduce plasma cholesterol, we performed 2-way ANCOVA analyses in which we controlled for the cholesterol-lowering capacity of different treatments. The square root was taken of the atherosclerotic lesion area to linearize the relationship with plasma cholesterol exposure as previously reported [38]. This involved dividing each individual value by the serum TC concentration, so that all groups began the test with identical serum TC concentrations. Unlike atorvastatin whose effect on atherosclerotic lesion area reduction was lost of significance after controlling for its cholesterol-lowering capacity, the effect of CGA on atherosclerotic lesion area reduction remained significant. This indicates that the reduction in atherosclerosis development upon atorvastatin treatment can be explained by its cholesterol-lowering effect, while additional mechanism(s) could be involved in the antiatherogenic effect of CGA.

To assess the potential additional antiatherosclerotic actions of CGA responsible for the protection against atherosclerosis, serum inflammatory parameters and cholesterol efflux from RAW264.7 macrophages were studied. Treatment with CGA significantly reduced the serum concentration of proinflammatory cytokines closely related with the development and progress of atherosclerosis such as IL-6, IL-8, TNFα and MCP-1 [39] (Figure 3) in ApoE^−/−^ mice and decreased the intracellular levels of IL-1β, IL-6 and TNFα in LPS-elicited RAW264.7 cells (Figure 4), indicating a potent antiinflammatory activity as previously reported [31], [40]. In contrast, atorvastatin showed non-significant effect on serum IL-6, IL-8 and TNFα (Figure 3). Supplementation with CGA also inhibited oxLDL-elicited macrophage foam cell formation (Figure 5) and stimulated NBD-cholesterol efflux to HDL and ApoA1 in RAW264.7 cells (Figure 6). Serum pharmacological experiments confirmed the physiological relevance of CGA with these *in vitro* effects on RAW264.7 macrophages (Figure 9). These data showed that CGA beneficially influences vascular inflammation and macrophage function, indicating that CGA may have local antiatherosclerotic effects in the vessel wall, which may explain its cholesterol-independent effect on atherosclerosis.

Stimulating efflux of cholesterol from macrophage is a critical way to inhibit macrophage foam cell formation and atherosclerotic lesion occurrence [3], [41]. ABCA1, ABCG1, LXRα and PPARγ are key regulators in cholesterol efflux. Treatment with CGA significantly increased the transcription of PPARγ, LXRα, ABCA1 and ABCG1 as determined by realtime quantitative PCR (Figure 7). The transcriptional activity of PPARγ was also increased by CGA in a dose dependent manner (Figure 8). The stimulating effect of CGA on cholesterol efflux in RAW264.7 macrophage was substantially abolished in the presence of specific PPARγ inhibitor GW-9662 (Figure 5). These results suggested that upregulation of the expression of PPARγ, LXRα, ABCA1 and ABCG1 and the transcriptional activity of PPARγ may participate in CGA-promoted cholesterol efflux from RAW264.7 cells.

As is well-known that CGA undergoes extensive metabolism after oral administration, it is therefore possible that the *in vivo* antiatherosclerotic effect of CGA may be mediated by its metabolites rather than CGA itself. It has been intensively reported that caffeic, quininic, ferulic, gallic and vanillic acids are main metabolites in plasma and urine after CGA administration [42]–[44]. To investigate the potential active compounds accounting for the *in vivo* effect of CGA, these five metabolites of CGA were tested for the effect on cholesterol efflux. Cholesterol efflux assay showed that caffeic, ferulic and gallic acids significantly stimulated cholesterol efflux from RAW264.7 cells mediated by HDL (Figure 10). The results were further confirmed by previous reports [44], [45]. These data suggested that caffeic acid, ferulic acid and gallic acid might be the potential active compounds accounting for the *in vivo* effect of CGA.

In conclusion, our results presented here demonstrate that CGA potently reduces atherosclerosis development in ApoE^−/−^ mice. The potential antiatherosclerotic actions of CGA responsible for the protection against atherosclerosis involve in decrease of serum lipid, suppression of vescular inflammation and promotion of cholesterol efflux from macrophages. Upregulation of PPARγ, LXRα, ABCA1 and ABCG1 transcription and PPARγ activity may involve in the stimulating effect of CGA on cholesterol efflux from macrophages. Caffeic acid, ferulic acid and gallic acid may be the potential active compounds accounting for the *in vivo* effect of CGA in animals.

## Supporting Information

Table S1 Oligonucleotide primers used in this work.
(DOC)
