# Supplementary material for: Chlorogenic Acid Protects against Atherosclerosis in ApoE−/− Mice and Promotes Cholesterol Efflux from RAW264.7 Macrophages
Source: PLoS One. 2014 Sep 4;9(9):e95452. doi: 10.1371/journal.pone.0095452 (PMC4154672; doi:10.1371/journal.pone.0095452)
Supplement: Table S1 — Oligonucleotide primers used in this work. (DOC) [file pone.0095452.s001.doc]

Table S1. Oligonucleotide primers used in this work

| Primers | Sequence (5’-3’) |
| --- | --- |
| PPARγ-F | CATTCTGGCCCACCAACTTC |
| PPARγ-R | TCAAAGGAATGCGAGTGGTCTT |
| LXRα-F | CCTTCCTCAAGGACTTCAGTTACAA |
| LXRα-R | CATGGCTCTGGAGAACTCAAAGAT |
| ABCA1-F | GCGGACCTCCTGGGTGTT |
| ABCA1-R | CAAGAATCTCCGGGCTTTAGG |
| ABCG1-F | AAGGCCTACTACCTGGCAAAGA |
| ABCG1-R | GCAGTAGGCCACAGGGAACA |
| β-actin-F | ACACTGTGCCCATCTACGAG |
| β-actin-R | CAGCACTGTGTTGGCATAGAG |
